# Diagnostic peritoneal lavage: a review of indications, technique, and interpretation

**DOI:** 10.1186/1757-7241-17-13

**Published:** 2009-03-08

**Authors:** Jill S Whitehouse, John A Weigelt

**Affiliations:** 1 Medical College of Wisconsin, Department of Surgery, 9200 W Wisconsin Ave, Milwaukee, WI 53226, USA; 2 Medical College of Wisconsin, Department of Surgery, Division of Trauma/Critical Care, 9200 W Wisconsin Ave, Milwaukee, WI 53226, USA

## Abstract

Diagnostic peritoneal lavage (DPL) is a highly accurate test for evaluating intraperitoneal hemorrhage or a ruptured hollow viscus, but is performed less frequently today due to the increased use of focused abdominal sonography for trauma (FAST) and helical computed tomography (CT). All three of these exams have advantages and disadvantages and thus each still play unique roles in the evaluation of abdominal trauma. Since DPL is performed less frequently today, a review of its indications, technique, and interpretation is pertinent.

## Introduction

Diagnostic peritoneal lavage (DPL) is an invasive, rapid, and highly accurate test for evaluating intraperitoneal hemorrhage or a ruptured hollow viscus. DPL plays a role in both blunt and penetrating abdominal trauma. First described in 1965, DPL replaced the four-quadrant abdominal tap, boasting a higher sensitivity and specificity in identifying intraabdominal injury [1]. Today DPL is performed less frequently, as it has been replaced by focused abdominal sonography for trauma (FAST) and helical computed tomography (CT). Yet, each of these diagnostic modalities has unique advantages and disadvantages.

DPL is the only invasive test of the three, but while lacking organ specificity it remains the most sensitive test for mesenteric and hollow viscus injuries [2,3]. FAST exams are rapid, noninvasive, and can be repeated multiple times throughout the resuscitation period. They are more user-dependent than DPL or CT scanning. Both FAST and DPL ineffectively evaluate retroperitoneal and diaphragmatic injuries and poorly identify solid organ injuries. Abdominopelvic CT scanning still requires a hemodynamically normal patient, is costly, and carries a small but significant lifetime risk of malignancy [4,5]. However, CT scanning reliably diagnoses solid organ injuries and evaluates the retroperitoneum, but its sensitivity and specificity for blunt bowel and mesenteric injuries is not superior to DPL [6]. As a result of these differences, all three tests continue to play important roles when evaluating a trauma patient for abdominal injuries.

Since DPL is performed less commonly today, a review of its indications, technique, and interpretation is pertinent.

## Indications

DPL is indicated in both blunt and a selective group of penetrating abdominal injuries. In blunt injuries, DPL has a number of indications but is dependent upon the patient's condition and availability of CT scanning and FAST. DPL is useful for patients who are in shock and when FAST capability is not available. Hypotensive patients should not be evaluated with CT scanning. In the absence of CT scanning, DPL is also useful in patients with an unreliable abdominal exam due to altered mental status or spinal cord injury. Other indications, when CT scanning is not available, include equivocal physical exam findings, the presence of a lap-belt sign, injuries to adjacent structures such as the lower ribs, lumbar spine, or pelvis, anticipated prolonged loss of contact with the patient (i.e. extraaabdominal procedures), or a high clinical suspicion of an intraabdominal injury.

The role of DPL in penetrating trauma is focused on patients with asymptomatic anterior abdominal stab wounds [7,8]. Patients with an anterior stab wound to the abdomen who are hemodynamically normal and have no signs of peritonitis are evaluated with a local wound exploration and if positive, a DPL is performed. Patients with flank wounds that track anteriorly are also candidates for DPL if the local wound exploration is positive [9].

The only absolute contraindication to DPL is previous abdominal surgery and this contraindication often is tempered by clinical judgment. The concern in these patients is that the DPL will actually injury an intra-abdominal organ when the catheter is introduced or that the fluid entrance and exit will be impeded by adhesions. Clinical judgment will allow some patients with previous abdominal surgery to be assessed with a DPL while in others the amount of surgery will clearly be a contraindication to DPL. Relative contraindications include preexisting coagulopathy, advanced cirrhosis, and morbid obesity. Relative contraindications to the standard infraumbilical approach include patients with a pelvic fracture or females beyond the 1^st ^trimester of pregnancy.

## Technique

DPL is performed one of three different ways [10,11]. The open technique utilizes a vertical infraumbilical incision and direct visualization of peritoneal entry with a scalpel. The closed technique relies on percutaneous needle access to the peritoneal cavity, followed by the insertion of a catheter using Seldinger technique. The semi-open technique follows the same principles of the open technique except that the midline fascia is penetrated with a needle and the catheter is advanced using the Seldinger technique. There is no difference in overall outcomes or rates of injury to visceral contents between the techniques [12-14]. The closed method is faster, but often has more technical complications such as wire placement and inadequate fluid return [12-14].

Regardless of the technique chosen, patient preparation is the same. First, the patient is positioned flat in the supine position. A Foley catheter and a nasogastric tube are inserted to decompress the bladder and stomach. The periumbilical area is surgically prepped and draped widely. A combination of local anesthesia and intravenous conscious sedation is used in hemodynamically normal patients. Local anesthesia alone will suffice in a hemodynamically abnormal patient. 1% lidocaine with epinephrine is used for local anesthesia to reduce the amount of cutaneous bleeding, which may lead to a false positive test.

**Figure 1:**
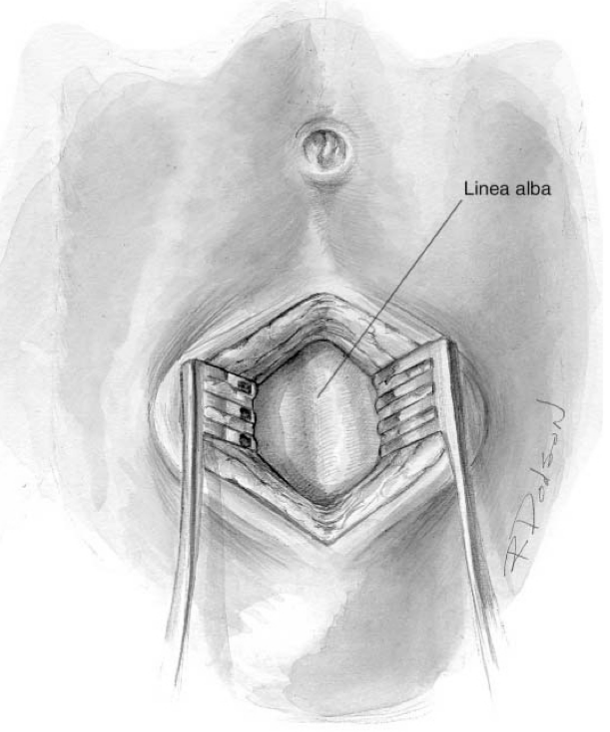
**View of the linea alba and anterior abdominal fascia following a midline infraumbilical incision for an open or semi-open approach to DPL**.

**Figure 2:**
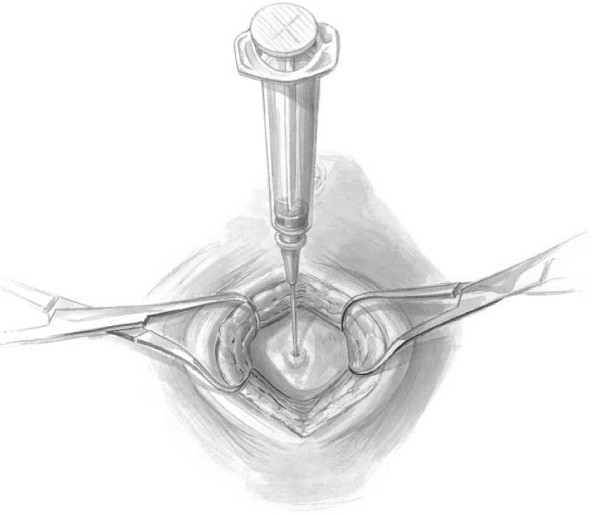
**While grasping and elevating the anterior abdominal fascia, an 18-guage needle is inserted at a 45-degree angle toward the pelvis**. Two "pops" are felt as the needle traverses the fascia and peritoneum.

**Figure 3:**
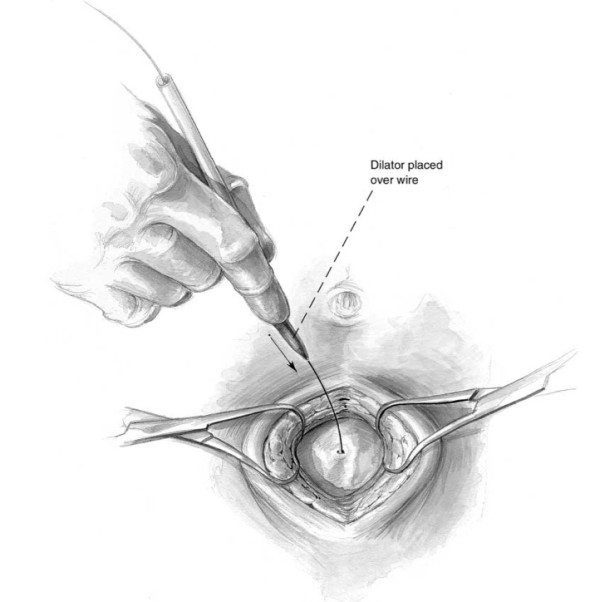
**Following guidewire placement through the needle, a dilator is passed through the fascia prior to placing the peritoneal catheter**.

The semi-open technique requires the periumbilical skin to be anesthetized and a vertical midline incision is made approximately 2 cm below or above the umbilicus [15]. Subcutaneous fat is dissected until the linea alba is identified (Figure 1). Retractors are placed to hold skin and subcutaneous tissue laterally. The fascia is grasped with two towel clips or hemostats on either side of the midline. An 18-guage needle is inserted at a 45-degree angle to the fascia toward the pelvis (Figure 2). As the needle successfully traverses the fascia and subsequent peritoneum, 2 "pops" are often felt. Filling the needle hub with saline as the catheter is advanced is helpful in detecting peritoneal penetration. The saline will flow through the needle as the peritoneal cavity is entered. A guidewire is passed through the needle into the pelvis. The wire should pass easily with no resistance. If the wire meets resistance, remove the needle and wire and start over. The needle is removed while keeping the wire stable. A dilator is passed over the wire and through the fascia and subsequently removed (Figure 3). Finally, the DPL catheter is introduced into the peritoneal cavity aimed toward the pelvis.

**Figure 4:**
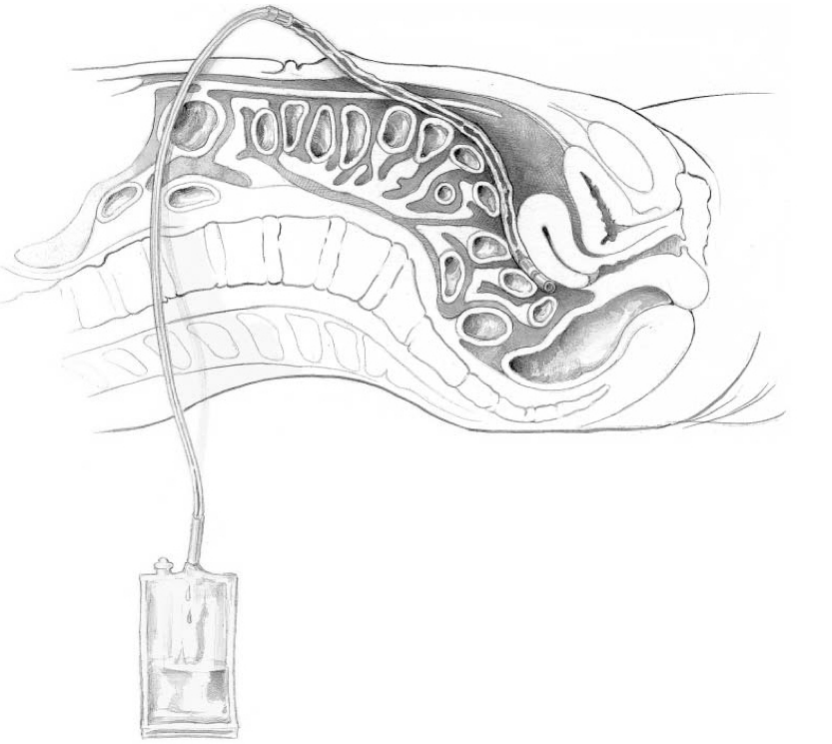
**After fluid is instilled, the bag is placed onto the floor to allow the intraabdominal fluid to return**. 30% of the original amount of instilled fluid is required for an adequate sample.

A syringe is used to aspirate the peritoneal contents. If blood flows easily into the syringe, most accept this as a positive aspirate and proceed with laparotomy. Others suggest 10 ml of blood constitutes a positive result [10]. In the absence of 10 ml blood, the DPL catheter is connected to a warmed liter bag of Lactated Ringers or normal saline using standard intravenous tubing. Care must be taken that the tubing has no one-way valves which would not allow fluid to flow freely back into the IV fluid bag. While the fluid infuses, gently rock the patient to allow mixing of the fluid with peritoneal contents. Once the bag is almost empty, place it on the floor and allow the intraabdominal fluid to return (Figure 4). Adequate fluid analysis requires at least 30% of the original amount infused. This usually amounts to 300–350 ml in an adult. In the pediatric patient, 10–15 ml/kg of fluid is infused and an adequate return is 20–30% of the total infusion [16]. This fluid is sent for gram stain and analysis of the red blood cell count and white blood cell count. It also should be grossly examined for enteric, bilious, or vegetable matter content. The wound is irrigated and only the skin requires surgical closure with either sutures or staples. If the open technique is used, the incised fascia should be closed. This stitch can be placed while the fluid is infusing and secured once the catheter is removed. If a closed technique is used then no stitch is required.

## Interpretation

A positive DPL in an adult classically requires one of the following results: 10 ml gross blood on initial aspiration, > 500/mm^3 ^white blood cells (WBC), > 100,000/mm^3 ^red blood cells (RBC), or the presence of enteric/vegetable matter [8]. These thresholds were originally developed in the setting of blunt trauma and have since been applied to penetrating trauma [1,17,18]. In the presence of gross blood or enteric matter, immediate laparotomy is performed. Without those findings, accurate cell counts should be obtained, which in our institution takes approximately 30 minutes to receive from the laboratory. During this time period, if the patient's clinical status deteriorates or signs of peritonitis develop, laparotomy is not delayed.

Some authors advocate lowering the threshold of RBCs in penetrating trauma to as low a 1,000 cells/mm, but others have shown significantly increased nontherapeutic procedure rates at lower thresholds [7,9,17-21]. Thacker reported an increase in the nontherapeutic celiotomy rate from 2.5% to 44% without a decrease in the number of missed injuries when 10,000 RBCs/mm^3 ^was used as the cutoff. Thal reported a comparable nontherapeutic procedure rate of 4.1% when 100,000 RBCs/mm^3 ^was used as the cutoff [7]. In the face of a 22% morbidity rate from negative laparotomies, one must be cognizant of the risk of lowering the threshold to operate [22].

**Figure 5:**
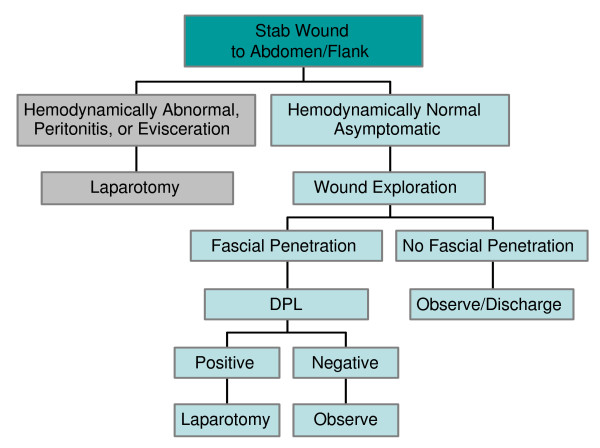
**Penetrating Trauma Algorithm**. Here, only stab wounds to abdomen and/or flank are considered, as DPL is not utilized in gunshot wounds. DPL is used in an asymptomatic patient with a positive wound exploration.

**Figure 6:**
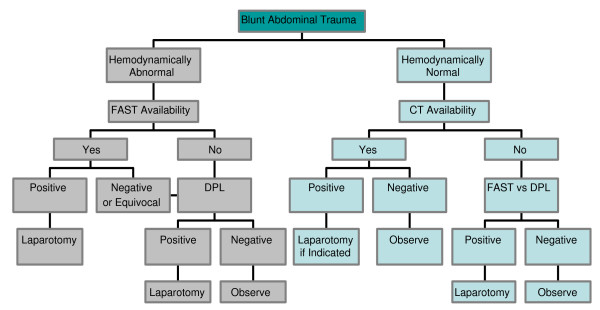
**Blunt Trauma Algorithm**. DPL is used when FAST and/or CT are not available. In a hemodynamically abnormal patient, if FAST is unavailable or results are equivocal, DPL is indicated. In a hemodynamically normal patient, DPL is used when CT and/or FAST are unavailable and the patient has concerning signs/symptoms of abdominal trauma.

In summary, adhering to > 100,000 RBCs/mm^3 ^as a marker of a positive DPL in both blunt and penetrating abdominal injuries is a safe and reliable practice. In penetrating stab wounds to the abdomen or flank, if the patient is hemodynamically abnormal or has signs of peritonitis, diagnostic testing should not delay laparotomy. In a hemodynamically normal, asymptomatic patient, DPL is used following a local wound exploration that reveals fascial penetration. FAST examination, when available, is helpful in the hemodynamically abnormal blunt trauma patient, but equivocal exams could be repeated or followed by a DPL. In the hemodynamically normal patient CT scanning is preferred given its non-invasive approach and accuracy. If CT is unavailable, either FAST or DPL should be used. Algorithms for using DPL, FAST, and CT scanning in both penetrating and blunt abdominal trauma are shown in Figures 5 and 6.

## Complications/follow-up

Patient safety is tantamount for all invasive procedures. Performing DPL safely is the goal. Most complications occur when principles are ignored. Not decompressing the urinary bladder or stomach increases the chances of injury to either organ with the DPL needle and catheter. In the obtunded patient, excessive pressure on the needle when entering the abdomen increases the likelihood of injury to the iliac vessels. When properly done, complication rates should be low. Two reports of over 2,500 DPLs each report an overall complication rate of 0.8%–1.7%, which included wound problems, inadequate fluid return, small bowel/mesenteric injuries, bladder punctures, and abdominal wall infusions [23,24].

Following a negative DPL, the wound should be monitored for infection. There is no evidence supporting prophylactic antibiotics unless indicated for a separate clinical condition. Non-absorbable sutures or skin staples placed at the time of closure are removed after 3–7 days either in the hospital or in a clinic setting following discharge.

## Competing interests

The authors declare that they have no competing interests.

## Authors' contributions

JSW performed the literature search and drafted the manuscript. JAW assisted with creating the algorithms presented and provided supervision of the manuscript writing. Both authors read and approved the final manuscript.
